# Supplementary material for: Validating the diagnostic performance of MUAC in screening moderate acute malnutrition and developing an optimal cut-off for under five children of different regions in Ethiopia
Source: PLoS One. 2022 Sep 29;17(9):e0273634. doi: 10.1371/journal.pone.0273634 (PMC9521901; doi:10.1371/journal.pone.0273634)
Supplement: Attachment — Submitted filename: Response to Reviewers.docx [file pone.0273634.s002.docx]

**Rebuttal letter that responds to each point raised by the academic editor and reviewers**

| Comment given by | Parts of Comment | Comments | Response by Author |
| --- | --- | --- | --- |
| By journal | General | Please ensure that your manuscript meets PLOS ONE's style requirements, including those for file naming.  Please include your tables as part of your main manuscript and remove the individual files.  Please clarify the sources of funding (financial or material support) | Accepted and corrected |
| By; Reviewer #1 | Title: | You should mention where these different regions are. | Accepted and corrected |
|  | Introduction | 1- In the line before the last one in the first paragraph you mentioned SAM please mention the full word. 2- You should define what is malnutrition and classification and degrees of it. 3- you should also mention the prevalence of malnutrition globally and the magnitude of the problem in your country 4- Please clarify; what did you mean by the sentence; " In addition to that the currently existing tools have low treatment outcome, increasing number of relapse and overburdening health system by relapse cases | 1. Accepted and corrected 2. Main topic about tools 3. Accepted and corrected |
|  | Methods | 1. u mentioned that you selected three different ethnicities, but you mentioned that one of the three regions which is Gambella has two different ethnic 2. In the paragraph started by Somali regional state: in the last line you mention the word (groups) twice so remove one of them. 3. you mentioned that, All apparently healthy under five children who were members of the community for more than six months were (include) in the study, please explain why did you define 6 months 4. you mentioned that, "For measuring weight hanging scale and seca Germany was used based on standard procedure", I think the sentence is not correct as this is the first time I hear about a hanging scale used for humans so please define which scale was used. 5. You mentioned also in the above paragraph, (heavy closes and shoes were removed). The word (closes) is incorrect in spelling, so remove it and put (clothes). 6. in the operational definitions; you defined wasting then you defined undernutrition as I think and as I understood from your the definitions they are the same so what did you mean by defining them separately | 1. they have collective names Nailo Saharans’ with the same origin. 2. Corrected and accepted. 3. Corrected and accepted. 4. Corrected and accepted 5. Accepted and corrected , thanks . 6. Clarification made for both readers and reviewers . |
|  | Results | 1- as regards the Nutritional status of under-five Children you need to revise the first paragraph. I think there are some words missing as you said, 2- "Regarding regional or ethnic differences in in the agreement of malnutrition by WHAZ and MUAC, a prevalence of 23.6% and 5.9% were observed by WHZ < - 2 Z and MUAC < 12.5 cm, respectively". I think you forgot to say that this was in Gambella. 3- Regarding Gender difference " Likewise, a prevalence of 17.3% and 6.2% had moderate wasting using WHAZ<-2 and MUAC<12.5cm, respectively" I think you forgot to mention that this prevalence was in females | 1. Accepted and corrected . 2. Accepted and corrected. 3. Accepted and corrected |
|  | Discussion | 1. In the sentence "In Gambella Region among Nuer and Anuak ethnic" replace the word (ethnic) with (ethnicity). 2. - in the sentence "Likewise, for Somalia Region the prevalence of MAM was 19.1% by WHZ<-2 and 8.6% by MUAC<12.5cm resulting in a misclassification of 6.6%." the misclassification is not = 6.6% as you mentioned but it's= 10.5%. 3. In the same in the sentence “The highest misclassification (17.7%) was observed in Gambella Region. 4. in the sentence "Somali ethnic group showing a faire agreement (Kappa=0.325) compare to gold standard WHZ<-2 score" use compared instead of compare. 5. the sentence "of the findings showed that the revised MUAC cut-off for the overall sample" you should not begin a sentence with a preposition. Moreover, the whole sentence needs reformulation 6. in the sentence " The difference in the optimal cut-of value for Somali region" correct the word (cut-of) repalc it by- (cut-off). 7. in the sentence "higher optimal cut-off (MUAC <18.85) developed in this study" you should correct the value of cut-off point vas you mentioned before it was (MUAC< 13.85) and not 18.85. | 1. Accepted and corrected 2. Accepted and corrected 3. Accepted and corrected 4. Accepted and corrected 5. Accepted and corrected 6. Accepted and corrected 7. Accepted and corrected |
|  | Conclusion: | needs reformulation | Accepted and corrected |
|  | Recommendation: | 1. in the beginning of the paragraph "The based on the findings" remove the word (The). 2. in the sentence "diverse ethnic" add the word (groups). 3. the sentence "different body composition, geographical and climate condition" use the words in plural; (compositions, conditions). | 1. Accepted and corrected. 2. Accepted and corrected. 3. Accepted and corrected. |
|  |  |  |  |
| By; Reviewer #2 | Abstract | It will be logical to spell out the MUAC abbreviation early in the abstract as some readers might not be aware of this. | Accepted and corrected. |
|  | Introduction: | The main aim of the study should be highlighted in the end as a separate brief paragraph. | Accepted and corrected. |
|  | Discussion | The limitations of the study should be clearly highlighted and the policy implications and future recommendations can also be further highlighted | Accepted and corrected. |
