# Supplementary material for: Validating the diagnostic performance of MUAC in screening moderate acute malnutrition and developing an optimal cut-off for under five children of different regions in Ethiopia
Source: PLoS One. 2022 Sep 29;17(9):e0273634. doi: 10.1371/journal.pone.0273634 (PMC9521901; doi:10.1371/journal.pone.0273634)
Supplement: Attachment — Submitted filename: Response _2_to Reviewers -.docx [file pone.0273634.s003.docx]

**Rebuttal letter that responds to each point raised by the academic editor and reviewers**

| Comment given by | Parts of Comment | Comments | Response by Author |
| --- | --- | --- | --- |
| By journal | General | If you would like to make changes to your financial disclosure, please include your updated statement in your cover letter. | No change made |
| By; Reviewer #2 | Abstract | It will be logical to spell out the MUAC abbreviation early in the abstract as some readers might not be aware of this. | Accepted and corrected. |
|  | Introduction: | The main aim of the study should be highlighted in the end as a separate brief paragraph. | Accepted and corrected. |
|  | Discussion | The limitations of the study should be clearly highlighted and the policy implications and future recommendations can also be further highlighted | Accepted and corrected. |
