# Supplementary material for: Validating the diagnostic performance of MUAC in screening moderate acute malnutrition and developing an optimal cut-off for under five children of different regions in Ethiopia
Source: PLoS One. 2022 Sep 29;17(9):e0273634. doi: 10.1371/journal.pone.0273634 (PMC9521901; doi:10.1371/journal.pone.0273634)
Supplement: Attachment — Submitted filename: Response _3_to Reviewers -.docx [file pone.0273634.s004.docx]

**Rebuttal letter that responds to each point raised by the academic editor and reviewers**

| Comment given by | Parts of Comment | Comments | Response by Author |
| --- | --- | --- | --- |
| By journal | General | Any changes to the reference list should be mentioned in the rebuttal letter that accompanies your revised manuscript. If you need to cite a retracted article, indicate the article’s retracted status in the References list and also include a citation and full reference for the retraction notice. | No change made |
| By; Reviewer #1: | Abstract | It will be logical to spell out the MUAC abbreviation early in the abstract as some readers might not be aware of this. | Accepted and corrected. |
|  | Introduction: | You have to notice that some readers may not be specialized so you should give an idea about malnutrition; its definition, classification, diagnosis and complications; even if if your main topic is about tools.  You didn't mention the prevalence of malnutrition globally and the magnitude of the problem in your country.  3- in line 99, (this study aimed to determine the diagnostic performance of MUAC for determination | Accepted and corrected. |
|  |  | The limitations of the study should be clearly highlighted and the policy implications and future recommendations can also be further highlighted  in line 100, please add the words (new) and (point) at the sentence to become (new optimal cut-off point | Accepted and corrected. |
|  | Methods | In line 128, you mentioned that the number of patients was (914) while you said that from each of the three regions you have done 305 cases so the total number must be (915).  2- in line 155, please add the word (scale) to become (Seca Germany Scale was used). | Accepted and corrected. |
|  | Result | In line 202, you should add the word (Approximately) to (half of them) as 50.8% is not exactly the half of cases.  2- as regards the Nutritional status of under-five Children you need to revise the first paragraph. I think there are some words missing as you said | Accepted and corrected |
