# Supplementary material for: Validating the diagnostic performance of MUAC in screening moderate acute malnutrition and developing an optimal cut-off for under five children of different regions in Ethiopia
Source: PLoS One. 2022 Sep 29;17(9):e0273634. doi: 10.1371/journal.pone.0273634 (PMC9521901; doi:10.1371/journal.pone.0273634)
Supplement: Attachment — Submitted filename: Response _4_to Reviewers - 4.docx [file pone.0273634.s005.docx]

**Rebuttal letter that responds to each point raised by the academic editor and reviewers**

| Comment given by | Parts of Comment | Comments | Response by Author |
| --- | --- | --- | --- |
| Reviewer #4: | General comment | - the same cut-off value of a body dimension can be reliable between 0-5 years of age for screening undernutrition status, please clarify it in the Introduction section that such a method, only one cut-off of MUAC can be used in this age interval. - It should also be clarified why MUAC and not weight to height cut-off or z-scores are suggested for underweight screening in children under the age of 5 in the studied region of Africa. - It is not clear how the use of MUAC cut-off value can distinguish between acute and chronic MAM. My suggestion is not to use the ‘acute’ attributive of MAM – either in the abstract or in the whole manuscript | - No modification; because age-specific MUAC is not developed yet By WHO or another organization and this issue is beyond the limit of the paper. - And the limitation for both suggestions were already addressed in line number 96,97 and 98 - No any change made; As far as my reading knowledge and as I am a nutrition department course provider there is no chronic MAM from the name itself it MAM is short form of Moderate acute malnutrition it is not chronic malnutrition. - In general MAM stands for moderate acute malnutrition no link with chronic under nutrition and chronic undernutrition is Stunting. |
| By; Reviewer #1: | Abstract | - A1 “Test variables is mid upper arm circumference (MUAC< 12.5cm).” – please correct this sentence, in this form it is not clear, too short, needs addition to help understanding. - A2: “In Amhara region there was fair agreement between the two measures in diagnosing MAM …” – please define MAM abbreviation on its first use. - A4: “WHZ<-2 for diagnosing MAM was …” – please first define WHZ abbreviation (also in the main manuscript). - I1: Line 73 – “Nutritional assessment can be done using anthropometric …” – change nutritional assessment to nutritional status assessment. | Accepted and corrected. |
|  | Main document | I1: Line 73 – “Nutritional assessment can be done using anthropometric …” – change nutritional assessment to nutritional status assessment. | Accepted and corrected in previous comments. |
|  | Discussion | The Discussion section is too long, please shorten it. | As the result is much if the discussions short it will affect the quality of the paper |
